# Combination treatment with 6-mercaptopurine and allopurinol in HepG2 and HEK293 cells – Effects on gene expression levels and thiopurine metabolism

**DOI:** 10.1371/journal.pone.0173825

**Published:** 2017-03-09

**Authors:** Sofie Haglund, Svante Vikingsson, Sven Almer, Jan Söderman

**Affiliations:** 1 Laboratory Medicine, Region Jönköping County, Sweden; 2 Department of Medicine, Solna, Karolinska Institutet, Stockholm, Sweden; 3 Division of Drug Research, Department of Medical and Health Sciences, Linköping University, Linköping, Sweden; 4 Center for Digestive Diseases, Karolinska University Hospital, Stockholm, Sweden; 5 Department of Clinical and Experimental Medicine, Linköping University, Linköping, Sweden; University of Crete, GREECE

## Abstract

Combination treatment with low-dose thiopurine and allopurinol (AP) has successfully been used in patients with inflammatory bowel disease with a so called skewed thiopurine metabolite profile. In red blood cells *in vivo*, it reduces the concentration of methylated metabolites and increases the concentration of the phosphorylated ones, which is associated with improved therapeutic efficacy. This study aimed to investigate the largely unknown mechanism of AP on thiopurine metabolism in cells with an active thiopurine metabolic pathway using HepG2 and HEK293 cells. Cells were treated with 6-mercaptopurine (6MP) and AP or its metabolite oxypurinol. The expression of genes known to be associated with thiopurine metabolism, and the concentration of thiopurine metabolites were analyzed. Gene expression levels were only affected by AP in the presence of 6MP. The addition of AP to 6MP affected the expression of in total 19 genes in the two cell lines. In both cell lines the expression of the transporter *SLC29A2* was reduced by the combined treatment. Six regulated genes in HepG2 cells and 8 regulated genes in HEK293 cells were connected to networks with 18 and 35 genes, respectively, present at known susceptibility loci for inflammatory bowel disease, when analyzed using a protein-protein interaction database. The genes identified as regulated as well as the disease associated interacting genes represent new candidates for further investigation in the context of combination therapy with thiopurines and AP. However, no differences in absolute metabolite concentrations were observed between 6MP+AP or 6MP+oxypurinol *vs*. 6MP alone in either of the two cell lines. In conclusion; the effect of AP on gene expression levels requires the presence of 6MP, at least *in vitro*. Previously described AP-effects on metabolite concentrations observed in red blood cells *in vivo* could not be reproduced in our cell lines *in vitro*. AP’s effects in relation to thiopurine metabolism are complex. The network-identified susceptibility genes represented biological processes mainly associated with purine nucleotide biosynthetic processes, lymphocyte proliferation, NF-KB activation, JAK-STAT signaling, and apoptotic signaling at oxidative stress.

## Introduction

Azathioprine and 6-mercaptopurine (6MP) are important drugs in the treatment of inflammatory bowel disease (IBD), including ulcerative colitis (UC) and Crohn’s disease (CD) [1–3].

Following oral administration the first pass metabolism of thiopurines is extensive (S1 Fig). Azathioprine is converted to 6MP in the presence of glutathione transferases or other sulfhydryl-containing proteins [4–6]. 6MP is then metabolized via enzymes of the purine metabolic and salvage pathway in nucleated cells. The main immunomodulating metabolites comprise the phosporylated thioguanine nucleotides (6TGNs) which are formed via hypoxanthine guanine phosphoribosyltransferase (HGPRT), inosine monophosphate dehydrogenase (IMPDH) and guanosine 5´-monophosphate synthetase (GMPS). Methylated thioinosine nucleotides (meTIN), formed via thiopurine S-methyltransferase (TPMT), also contribute to the immunomodulatory effects [7–11].

It is considered that up to 50% of patients are either intolerant of refractory to standard thiopurine therapy [12]. A skewed metabolite profile with excessive production of meTIN and subtherapeutic 6TGN levels is found in approximately 15–20% of patients [13, 14] and has been associated with glucocorticosteroid dependency and adverse events [15–17]. Thus it seems that the dominant metabolic pathway may differ between patient groups. The underlying mechanism to why a proportion of patients preferentially metabolize azathioprine and 6MP to meTIN is currently unknown. Combination treatment with allopurinol, a xanthine oxidase (XO) inhibitor, and a reduced dose of thiopurine (~25–33% of original dose) has successfully been used in these patients and switches the metabolism towards predominately 6TGN production and improved therapeutic efficacy [15, 18]. However, high XO activity *per se* does not explain the phenotype [19].

In clinical practice, monitoring of thiopurine metabolites in red blood cells (RBC) is used as a surrogate compartment for mononuclear cells, the target cells of therapy, and it is generally appreciated that 6TGNs are synthesized via IMPDH. However, IMPDH is known to be essentially non-functional in RBC [20, 21] and XO is considered absent in circulating blood cells in general [20, 22]. Possibly RBC synthesize 6TGNs from thiopurine bases or nucleosides produced via hepatic or other tissue metabolism [21, 23]. Thus, AP probably mediates its effect on the thiopurine metabolism and RBC metabolite concentrations via several mechanisms, not only via XO. It would therefore be interesting to study the effect of AP on thiopurine metabolism in cells with an active pathway for the synthesis of 6TGN.

Our aims were to elucidate the effects of AP on gene expression levels and thiopurine metabolism under controlled conditions in a single biological compartment (compared to the situation in RBC) using two cell lines; the liver cell line HepG2 +/- transiently transfected to express XO, and the human embryonic kidney cell line HEK293 (not expressing XO). These cell lines are functionally well characterized, they express most of the genes of known relevance to thiopurine metabolism that are not operating in RBC, they are DNA mismatch repair proficient, considered important for thiopurine toxicity, and have previously been used by several groups in studies of the thiopurine metabolism [24–32].

Here we describe new candidate genes worth investigating further in the context of combination therapy with thiopurines and AP. The previously described AP-effect on metabolite concentrations observed in RBC *in vivo* was not reproduced in our cell lines.

## Material and methods

### Ethics statement

No ethics committee approval was required for this study as all experiments were conducted using established commercial cell lines.

### HepG2 cells: Transfection and incubation with drugs

The *E*. *coli* DHB10 strain containing the Gateway ptREX-DEST30 vector with the cDNA encoding XO (BC166696) was from ImgaGenes (Berlin, Germany) and was propagated and enriched according to the manufacturer’s instructions. Plasmids were isolated with the S.N.A.P Plasmid DNA Midi kit (Life Technologies, Carlsbad, CA, USA).

Fetal calf serum (FCS), Lipofectamine 2000, and Opti-MEM were from Life Technologies. Pencillin-streptomycin, 6MP, AP, and oxypurinol were from Sigma Aldrich (St Louis, MO, USA).

HepG2 cells (ATCC^®^ HB-8065, LGC standards, Teddington, UK) were maintained in Eagle’s minimum essential medium (LGC standards) supplemented with 10% FCS, and penicillin-streptomycin (100 U mL^-1^ resp. 100 μg mL^-1^) at 37°C in a humidified atmosphere with 5% CO_2_. Cells were grown in 6-well trays (0.2x10^6^ cells per well) overnight in medium without antibiotics before experiments were started. Thereafter 2 μg plasmid was mixed with Optimem and Lipofectamine 2000 and transfection was performed according to the manufacturer´s instructions. Cells not transfected to express XO were not MOCK-transfected as comparisons were made within each condition (i.e. +/-XO). Drugs [6MP (6 μM), AP (100 μM) or the combination of 6MP+AP] were dissolved in 0.1 M NaOH, diluted in growth medium and added to the cell cultures grown overnight. Control cultures received the same concentration of solvent.

### HEK293 cells: Cell culture and incubation with drugs

The EcRHEK293 cell line (Invitrogen, Carlsbad, CA, USA) was a gift from Dr Sally Coulthard (The Institute of Cellular Medicine, Newcastle University Medical School, Newcastle upon Tyne, UK). However, the described inducible promoter-system for TPMT in this cell line [28] was not used. Medium and antibiotics were from Life Technologies. Cells were maintained in Dulbecco’s medium supplemented with 10% heat-inactivated FCS, geneticine (500 μg mL^-1^) and zeocin (400 μg mL^-1^), at 37°C in a humidified atmosphere with 5% CO_2_. Cells [2x10^6^ cells per 60 cm^2^ vials] were grown overnight in medium without antibiotics before the addition of drugs [6MP (3 μM), AP (100 μM), oxypurinol (100 μM) or the combination of 6MP+AP or 6MP+oxypurinol]. Drugs were dissolved in 0.1 M NaOH and diluted in growth medium. Control cultures received the same concentration of solvent. In HEK293 cells oxypurinol was added since these cells, in contrast to HepG2 cells, express only low levels of *AOX1*, considered important in the conversion of AP to its metabolite oxypurinol [33, 34].

### Additional procedures

Both cell lines were checked for misidentification or contamination in the the ICLAC Database of Cross-contaminated or Misidentified Cell Liners (version 7.2 released 14 October, 2014). All drug concentrations were selected not to exceed the observed mean plasma and tissue concentrations *in vivo* after therapeutic doses [30, 34]. The selected concentration of 6MP corresponds to the IC_50_-value (50% inhibitory concentration on cell growth) in HEK293 cells [27] whereas 6MP up to 4 mM is expected to be non-toxic to HepG2 cells [24].

After the addition of drugs, HepG2 and HEK293 cells were grown for 73 h and then harvested, washed twice in cold phosphate buffered saline (PBS) pH 7.4 and counted manually. For HepG2 cells, one well per treatment were used for isolation of RNA, whereas an aliquot of cells was taken from each setting in the HEK293 cells. Cells were washed once with PBS before 350 μL buffer RLT plus (Qiagen, Hilden, Germany) was added. Cells were homogenised, and RNA isolated with the RNeasy PLUS mini kit (Qiagen). All experiments were repeated three times.

### Measurement of IMPDH activity in HEK293 cells

Based on its position in the metabolic scheme of thiopurines blockage of IMPDH may explain a high meTIN/6TGN ratio *in vivo* and induction of this enzyme could restore 6TGN. We therefore investigated the effect of drugs on the enzyme activity of IMPDH in HEK293 cells.

Cells (approximately 10x10^6^ cells mL^-1^) from each experiment were lysed in water by two freeze thaw cycles. IMPDH activity was measured by high performance liquid chromatography (HPLC) as described previously [35, 36].

### Thiopurine metabolites in HepG2 and HEK293 cells

Dithioerythritol (DTE), perchloric acid, 6TG, 6MP, thioxanthine (TX) and 6-methyl-MP were obtained from Sigma Aldrich. Standards of thioguanosine monophosphate (TGMP) and methyl thioinosine monophosphate (meTIMP) were from Jena BioScience (Jena, Germany).

#### HepG2 cells

The concentration of meTIN, 6TGN, TIMP, and the sum of TXMP and TX, were determined as their corresponding bases 6-methyl-MP, 6TG, 6MP and TX with IP-RP-HPLC. Pellets of approximately 2x10^6^ cells were prepared as duplicates from each experiment and immediately frozen. The cell pellets were re-suspended in MilliQ water and lysed by sonication in an ice cold water bath for 15 min followed by 15 min of centrifugation at 17 530 x g at 4°C. Sixty μL of supernatant was mixed with 40 μL of 130 mM DTE in 17% perchloric acid (w/v) and vortexed for 5 minutes followed by 3 minutes centrifugation at 10.000 x g at 4°C. Ninety μL of supernatant was boiled at 100°C for 45 minutes and thereafter placed on ice and diluted with 45 μl MilliQ water. Fifthy μL of the sample was injected onto an HPLC system consisting of a Phenomenex Synergi MaxRP column 150x2 mm (4 μm, cooled to 10°C), a Dual λ Absorbance Detector 2487 (Waters, Sollentuna, Sweden) and a 2695 Separations Module pump (Waters). The mobile phase delivered isocratically at 0.45 mL min^-1^ consisted of 0.02 M phosphoric acid, 1.3 mM DTE, 0.75% acetonitrile and 0.25% methanol (v/v). 6TG and TX were detected at 340 nm, 6-methyl-MP at 290 nm and 6MP at 325 nm. Total run time was 15 minutes. Eight calibration standards per analyte were used; 6TG: 27–5330, 6-methyl-MP: 267–53300, 6MP: 40–8000, TX: 67–13300 pmol mL^-1^ in a 60 μL blank cell lysate. Low (160% of lowest calibrator, inter-batch CV <10% and accuracy between 90–111% for all analytes) and high (75% of highest calibrator, inter-batch CV <5% and accuracy 97–107% for all analytes) quality control samples were included in each run.

#### HEK293 cells

Pellets of approximately 4x10^6^ cells were prepared as duplicates from each experiment and immediately frozen. The concentrations of meTIMP and 6TGMP were measured in HEK293 cells by HPLC as previously described [27] with the following modifications to allow protein concentration measurements: cell pellets were re-suspended in 100 μL MilliQ water and lysed by sonication on ice. Twenty μL was taken to protein concentration measurement and 70 μL of the cell lysate was mixed with 90 μL of 40 μM 6-ethylmercaptopurine solution (internal standard) followed by 400 μL of 1.6 mM EDTA in 97% acetonitrile to precipitate the proteins. Cells were then derivatized and analyzed.

Metabolite concentrations from both cell lines were normalized to the protein concentration of the cell lysate as determined with the Pierce^™^ BCA^™^ Protein Assay (Life Technologies) and expressed as pmol mg protein^-1^.

### Gene expression analysis

Genes previously associated with the RBC concentration of thiopurine metabolites and/or the meTIN/6TGN concentration ratio when gene expression levels were studied in whole blood of patients with IBD [37] were included in this study. The mRNA expression of 49 target genes including *AOX1* and *MOCOS* was analyzed in HepG2 cells and 46 target genes in HEK293 cells (S1 Table). The lower number of genes in HEK293 cells was due to low expression in previous experiments and to logistic issues.

RNA concentration was assessed with Nanodrop^®^ ND-1000 spectrophotometer (Nanodrop Technologies, Wilmington, DE) and RNA integrity with 2100 Bioanalyzer (Agilent technologies, Santa Clara, CA).

Real-time PCR was performed with the FAST 7500 real-time PCR system and reagents from Life Technologies with 5–10 ng cDNA per reaction in a final volume of 10 μL. Amplification curves were evaluated and the C_T_-values (threshold cycle) estimated in ExpressionSuite software v 1.0 (Life Technologies).

In both cell lines, nine potential reference genes were evaluated for low sample-to-sample variation across the different experimental conditions using the Normfinder algorithm [38]. Finally, *YWHAZ* was selected in HepG2 cells whereas *POP4* and *ACTB* were selected in HEK293 cells.

Gene expression was normalized against the expression level of the reference genes in Genex Professional software version 4.3.8 (MultiD Analysis AB, Göteborg, Sweden) to obtain a delta-C_T_ (dC_T_). The relative expression (RQ) was determined for each gene in relation to the sample with the lowest expression (highest C_T_).

### Data analysis

#### Statistics

For group comparisons two-sided t-tests were used and *P*-values were corrected for multiple testing according to Benjamini-Hochberg [39]. One-way ANOVA with Unequal N HSD post hoc test was applied when evaluating IMPDH activity in HEK293 cells. Data are expressed as mean ± SD or range (min-max). Statistical analyses were performed using Statistica version 12.7 (StatSoft Inc, Tulsa, OK, USA). Results were considered significant if corrected *P*-values were < 0.05.

#### Pathway analyses

Under the assumption that proteins encoded by AP regulated genes may interact with and affect other proteins, a protein-protein interaction analysis was performed. Genes identified as regulated by drug treatment were evaluated for protein-protein interactions with prioritized genes present at susceptibility loci identified for CD, UC and IBD overall [40] with the Search Tool for the Retrieval of Interacting Genes/Proteins database, STRING, version 10.0 [41] as previously described [37]. The interacting IBD susceptibility candidate genes were tested for enrichment in GeneOntology terms associated with biological processes using the PANTHER over-representation test (release 2016-07-15) [42] and the GeneOntology database (release 2016-09-24). Bonferroni correction for multiple testing was applied and results were considered significant if corrected *P*-values were < 0.05.

## Results

### Gene expression levels in HepG2 cells

**Fig 1:**
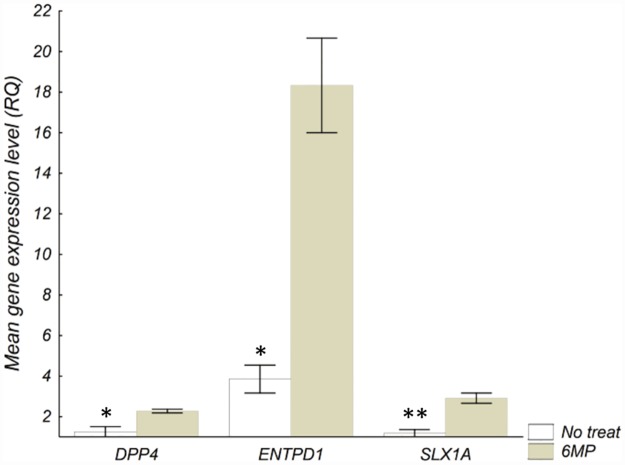
Genes affected by 6MP in HepG2 cells not transfected to express XO. Genes differently expressed when comparing incubation with 6MP (6 μM) with medium (no treat) in HepG2 cells not transfected to express XO. Cells were incubated with drug for 73 h before isolation of RNA and analysis. Values are presented as mean of three experiments ± SD. Differences were compared with the two-sided t-test and *P*-values were corrected for multiple testing. 6MP 6-mercaptopurine, XO xanthine oxidase, RQ relative gene expression, *P < 0.*05*, **P < 0.*01*.

**Fig 2:**
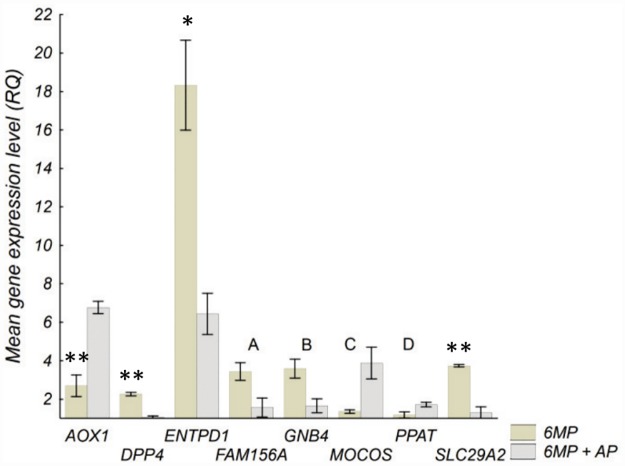
Gene expression in HepG2 cells not transfected to express XO after combined treatment with 6MP+AP. Genes differently expressed when comparing incubation with 6MP (6 μM) +AP (100 μM) with 6MP alone (6 μM) in HepG2 cells not transfected to express XO. Cells were incubated with drugs for 73 h before isolation of RNA and analysis. Values are presented as mean of three experiments ± SD. Differences were compared with the two-sided t-test and *P*-values were corrected for multiple testing. 6MP 6-mercaptopurine, AP allopurinol, XO xanthine oxidase, RQ relative gene expression, A; *P* = 0.06, B; *P* = 0.05, C; *P* = 0.05, D; *P* = 0.05, * P < 0.05, ** P < 0.01.

In HepG2 cells, 6MP resulted in an up-regulation of *DPP4*, *ENTPD1*, and *SLX1A* (Fig 1). With the combined treatment (6MP+AP), compared with 6MP alone, cells expressed reduced levels of *DPP4*, *ENTPD1*, *FAM156A*, *GNB4* and *SLC29A2*, and increased levels of *AOX1*, *MOCOS* and *PPAT* (Fig 2). No genes were regulated by AP alone. In HepG2 cells transfected to express XO, no genes were regulated by AP, 6MP or the combination treatment.

### Gene expression levels in HEK293 cells

**Fig 3:**
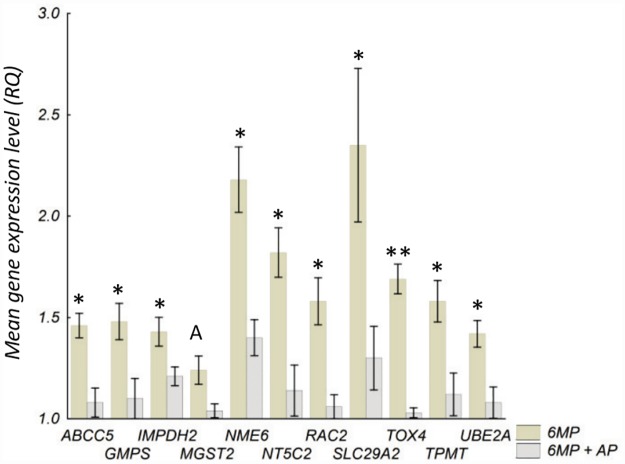
Gene expression in HEK293 cells after combined treatment with 6MP+AP. Genes differently expressed when comparing incubation with 6MP (3 μM) +AP (100 μM) with 6MP alone (3 μM) in HEK293 cells. Cells were incubated with drugs for 73 h before isolation of RNA and analysis. Values are presented as mean of three experiments ± SD. Differences were compared with the two-sided t-test and *P*-values were corrected for multiple testing. 6MP 6-mercaptopurine, AP allopurinol, RQ relative gene expression, A; *P* = 0.05, * P < 0.05, ** P < 0.01.

In HEK293 no genes were affected by 6MP alone. When AP was added to 6MP, cells down-regulated the expression levels of *ABCC5*, *GMPS*, *IMPDH2*, *MGST2*, *NME6*, *NT5C2*, *RAC2*, *SLC29A2*, *TOX4*, *TPMT* and *UBE2A* compared with 6MP alone (Fig 3). In cells treated with oxypurinol the relative expression of *CTSS* increased [RQ 1.37 (1.17–1.56) to 2.71 (2.56–2.92), *P* < 0.05] as did the relative expression of *TUSC2* [RQ 1.10 (1.06–1.15) to 1.83 (1.72–2.01), *P* < 0.05]. However, no genes were regulated by the combination treatment of 6MP+oxypurinol compared with 6MP alone or by AP alone.

### Pathway analyses

#### HepG2 cells

**Fig 4:**
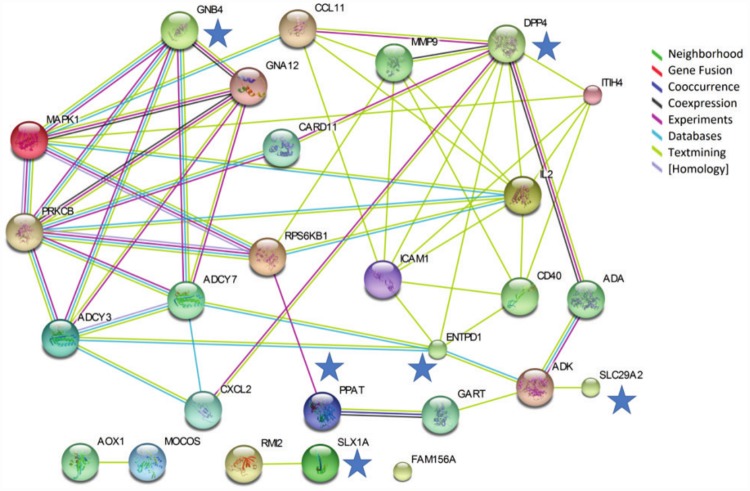
Protein-protein network analysis based on regulated genes in HepG2 cells. Interactions between the nine genes identified as regulated by 6MP (6 μM) or 6MP (6 μM) +AP (100 μM) compared with 6MP alone in HepG2 cells not transfected to express XO, and IBD susceptibility candidate genes. The interactions were identified with the Search Tool for the Retrieval of Interacting Genes/Proteins database. Blue stars indicate the 6 investigated genes which interacted with 18 IBD susceptibility candidate genes. 6MP 6-mercaptopurine, AP allopurinol, XO xanthine oxidase, IBD inflammatory bowel disease.

Six (*DPP4*, *ENTPD1*, *GNB4*, *PPAT*, *SLC29A2*, *SLX1A*) of 9 genes regulated by the presence of 6MP and/or the combination therapy (6MP+AP) compared with 6MP alone interacted with 18 genes present at 17 IBD susceptibility loci associated with CD (n = 2), UC (n = 4) and IBD overall (n = 11) as judged by the STRING analysis (Fig 4 and Table 1).

**Table 1:** IBD susceptibility loci associated with interacting IBD susceptibility candidate genes in the protein-protein network analysis comprising HepG2 cells.

Risk loci	Interacting gene	Disease
rs2066847	*ADCY7*	CD
rs2284553	*GART*	CD
rs6545800	*ADCY3*	IBD
rs2227564	*ADK*	IBD
rs30913116	*CCL11*	IBD
rs1569723	*CD40*^1^	IBD
rs2472649	*CXCL2*	IBD
rs11879191	*ICAM1*	IBD
rs7657746	*IL2*	IBD
rs2266959	*MAPK1*	IBD
rs1569723	*MMP9*^1^	IBD
rs7404095	*PRKCB*	IBD
rs529866	*RMI2*	IBD
rs1292053	*RPS6KB1*	IBD
rs6017342	*ADA*	UC
rs798502	*CARD11*	UC
rs798502	*GNA12*	UC
rs9847710	*ITIH4*	UC

Six of nine genes identified as regulated by 6MP (6 μM) or the combination 6MP+AP (100 μM) compare with 6MP alone in HepG2 cells not transfected to express XO, interacted with 18 IBD susceptibility candidate genes when evaluated with the Search Tool for the Retrieval of Interacting Genes/Proteins database. The table lists the IBD susceptibility loci associated with each IBD susceptibility candidate gene.

^1^Genes linked to the same risk loci.

6MP 6-mercaptopurine, AP allopurinol, XO xanthine oxidase, CD Crohn’s disease, UC ulcerative colitis, IBD inflammatory bowel disease.

The 18 network-identified susceptibility candidate genes were significantly enriched in 84 GeneOntology terms associated with biological processes representing mainly B-cell proliferation/activation, purine nucleotide biosynthetic related processes, proliferation of monocytes/lymphocytes, as well as NF-KB activation (S2 Table).

#### HEK293 cells

**Fig 5:**
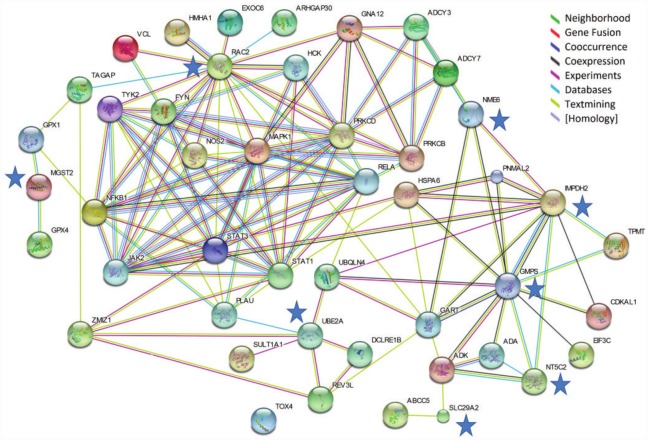
Protein-protein network analysis based on regulated genes in HEK293 cells. Interactions between the eleven genes identified as regulated by 6MP (3 μM) +AP (100 μM) compared with 6MP alone in HEK293 cells, and IBD susceptibility candidate genes. The interactions were identified by the Search Tool for the Retrieval of Interacting Genes/Proteins database. Blue stars indicate the 8 genes which interacted with 35 IBD susceptibility candidate genes. 6MP 6-mercaptopurine, AP allopurinol, IBD inflammatory bowel disease.

Eight (*GMPS*, *IMPDH2*, *MGST2*, *NME6*, *NT5C2*, *RAC2*, *SLC29A2*, *TPMT*) out of the 11 genes regulated by the combination therapy (6MP+AP) interacted with 35 genes present at 32 susceptibility loci associated with CD (n = 9), UC (n = 4) and IBD overall (n = 19), as judged by the STRING analysis (Fig 5 and Table 2). Out of 35 interactions, 17 were uniquely related to *RAC2*. *TOX4* did not show up in any network and *ABCC5* and *TPMT* interacted only with genes included in the RT qPCR analyses; *SLC29A2* and *GMPS* and *IMPDH2*, respectively. The 35 network-identified susceptibility genes were significantly enriched in 84 GeneOntology terms associated with biological processes representing mainly the JAK-STAT cascade involved in growth hormone receptor signaling, apoptotic signaling in response to oxidative stress, response to hydrogen peroxide, as well as platelet activation and purine nucleotide biosynthetic processes (S2 Table).

**Table 2:** IBD susceptibility loci associated with interacting IBD susceptibility candidate genes in the protein-protein network analysis comprising HEK293 cells.

Risk loci	Interacting gene	Disease
rs2066847	*ADCY7*	CD
rs6679677	*DCLRE1B*	CD
rs2284553	*GART*	CD
rs2024092	*GPX4*^1^	CD
rs2024092	*HMHA1*^1^	CD
rs2945412	*NOS2*	CD
rs4802307/rs1126510	*PNMAL2*	CD
rs212388	*TAGAP*	CD
rs6545800	*ADCY3*	IBD
rs2227564	*ADK*^2^	IBD
rs4656958	*ARHGAP30*	IBD
rs26528	*EIF3C*^3^	IBD
rs7911264	*EXOC6*	IBD
rs3851228	*FYN*^4^	IBD
rs3197999	*GPX1*	IBD
rs6142618	*HCK*	IBD
rs1801274	*HSPA6*	IBD
rs10758669	*JAK2*	IBD
rs2266959	*MAPK1*	IBD
rs2227564	*PLAU*^2^	IBD
rs7404095	*PRKCB*	IBD
rs7608910	*RELA*	IBD
rs3851228	*REV3L*^4^	IBD
rs1517352	*STAT1*	IBD
rs12942547	*STAT3*	IBD
rs26528	*SULT1A1*^3^	IBD
rs11879191	*TYK2*	IBD
rs670523	*UBQLN4*	IBD
rs2227564	*VCL*^2^	IBD
rs1250546	*ZMIZ1*	IBD
rs9358372/rs12663353	*CDKAL1*	IBD/CD
rs6017342	*ADA*	UC
rs798502	*GNA12*	UC
rs3774959	*NFKB1*	UC
rs9847710	*PRKCD*	UC

Eight of eleven genes identified as regulated by the combination 6MP (3 μM) + AP (100 μM) compare with 6MP alone in HEK293 cells interacted with 35 IBD susceptibility candidate genes when evaluated with the Search Tool for the Retrieval of Interacting Genes/Proteins database. The table lists the IBD susceptibility loci associated with each IBD susceptibility candidate gene.

^1-4^Genes linked to the same risk loci.

6MP 6-mercaptopurine, AP allopurinol, CD Crohn’s disease, UC ulcerative colitis, IBD inflammatory bowel disease.

### IMPDH activity in HEK293 cells

IMPDH activity was measured in HEK293 cells. There was no measurable difference in the IMPDH activity when 6MP (115 nmol mg protein^-1^ h^-1^; range 112–118, *P* = 0.78) or AP (104 nmol mg protein^-1^ h^-1^; 97–113, *P* = 1.00) was added compared with untreated cells (103 nmol mg protein^-1^ h^-1^; 101–108). Similarly, the combination treatment of 6MP+AP (133 nmol mg protein^-1^ h^-1^; 114–157) or 6MP+oxypurinol (126 nmol mg protein^-1^ h^-1^; 120–133) did not affect the IMPDH activity compared with 6MP alone (*P* = 0.44 and 0.91, respectively).

### Concentration of thiopurine metabolites in HepG2 cells

**Fig 6:**
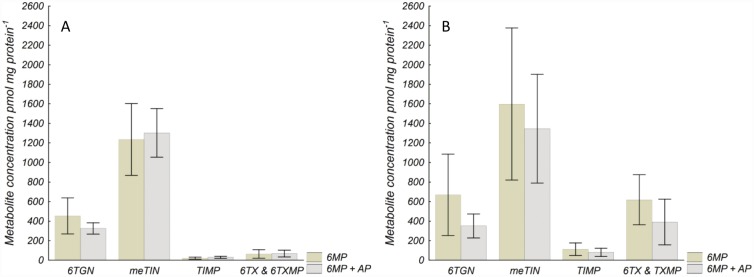
Concentration of thiopurine metabolites in (A) HepG2 cells not transfected to express XO and in (B) HepG2 cells transfected to express XO. Concentration of thiopurine metabolites in HepG2 cells incubated with 6MP (6 μM) or the combination 6MP (6 μM) + AP (100 μM) for 73 h. Metabolites were measured by HPLC. Values are mean ± SD of three experiments. No differences in absolute metabolite concentrations were observed between the two conditions when compared with the two-sided t-test and *P*-values were corrected for multiple testing. 6MP 6-mercaptopurine, AP allopurinol, 6TGN thioguanine nucleotides, meTIN methylated thioinosine nucleotides, TIMP thioinosine monophosphate, 6TX thioxanthine, 6TXMP thioxanthosine monophosphate.

No differences in absolute metabolite concentrations were observed between 6MP+AP *vs*. 6MP in HepG2 cells, irrespective of transfection for XO expression or not (Fig 6A and 6B). However, the meTIN/6TGN concentration ratio increased from 2.9 (range 2.5–3.8) to 4.0 (range 3.6–4.4) by the combination treatment compared with 6MP alone in HepG2 cells not expressing XO (*P* < 0.05), but was unaffected in HepG2 cells expressing XO [combination treatment 3.8 (3.2–4.3) *vs*. 6MP alone 2.7 (range 2.1–4.0); *P* = 0.25].

### Concentration of thiopurine metabolites in HEK293 cells

**Fig 7:**
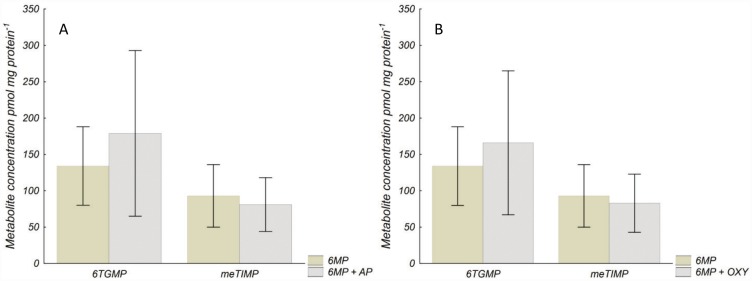
Concentration of thiopurine metabolites in (A) HEK293 after incubation with 6MP and 6MP+AP, and in (B) HEK293 cells after incubation with 6MP and 6MP+oxypurinol. Concentration of thiopurine metabolites in (A) HEK293 cells incubated with 6MP (3 μM) or the combination 6MP (3 μM) +AP (100 μM) for 73 h. (B) Concentration of thiopurine metabolites in HEK293 cells incubated with 6MP (3 μM) or the combination 6MP (3 μM) + oxypurinol (100 μM) for 73 h. Metabolites were measured by HPLC. Values are mean ± SD of three experiments. No differences in absolute metabolite concentrations were observed between the two conditions when compared with the two-sided t-test and *P*-values were corrected for multiple testing. 6MP 6-mercaptopurine, AP allopurinol, OXY oxypurinol, 6TGMP thioguanosine monophosphate, meTIMP methyl thioinosine monophosphate.

In HEK293 cells no differences in metabolite concentrations were observed between 6MP+AP or 6MP+oxypurinol *vs*. 6MP alone (Fig 7A and 7B). The metabolite concentration ratio was not affected by the combination treatment compared with 6MP alone [6MP+AP 0.5 (0.4–0.7), 6MP+oxypurinol 0.6 (0.4–1.0), 6MP alone 0.8 (0.4–1.1); *P* = 0.39 and 0.96].

## Discussion

Here we investigated the effects of the addition of AP to 6MP in cell-based models (HepG2 and HEK293 cells) with active thiopurine metabolic pathways and in which both gene expression levels and metabolite concentrations were studied following exposure to clinically relevant drug concentrations.

In total, 19 genes which were regulated when AP was added to 6MP in the two cell lines were identified. Six out of nine regulated genes in HepG2 cells and eight out of eleven regulated genes in HEK293 cells participated in interaction networks with 45 genes from 42 susceptibility loci for CD, UC or IBD overall, present among the 163 susceptibility candidate genes identified by Jostins et al. [40].

Expression levels of investigated genes were affected by AP only in the presence of 6MP and in cells not expressing XO. The expression level of *SLC29A2* was down-regulated by the combined treatment (6MP+AP) compared with 6MP alone both in HepG2 cells and in HEK293 cells. *SLC29A2* encodes the equilibrate nucleoside transporter 2, ENT2. This family of proteins (SLC29) transports purine and pyrimidine nucleosides as well as nucleobases downward a concentration gradient over the plasma membrane [43]. It is possible that AP can affect the transport and intracellular concentration of 6MP (or its metabolites) via this transporter, but the substrate affinities are unknown. *SLC29A2* interacted with *ADK* in the network analysis. *ADK* encodes adenosine kinase which phosphorylates the thiopurine metabolite meMP-riboside to generate meTIMP [44]. In MOLT4 cells down-regulation of *SLC29A2* is associated with reduced influx of 6MP and less cytocidal effects [45]. However, metabolites were not measured after down-regulation. Both the ENT2 transporter and adenosine kinase also have other important roles such as regulating adenosine levels, of importance for many immunoregulatory processes [46].

In HepG2 cells, the expression levels of both *ENTPD1* and *DPP4* were induced by 6MP, followed by a reduced expression with the combined treatment (6MP+AP). The liver synthesizes most of the nucleotides in the body and purinergic signaling regulates many hepatic processes. *ENTPD1* encodes ectonucleoside triphosphate diphosphohydrolase 1 (also known as CD39) which can hydrolyze ATP and other nucleotides to regulate the extracellular purinergic turnover. Reduced ATP-hydrolysis may potentially affect the activities of ATP-driven drug transporters such as MRP4 and MRP5 (encoded by *ABCC5*, down-regulated in HEK293 cells), studied here, as well as MRP8 and MRP9 [32], but also the activity of kinases required for maintaining the intracellular pool of nucleoside monophosphates. Altered expression of *ENTPD1* also impact hepatic metabolism, inflammation and immunity [47]. *DPP4* encodes dipeptidyl-peptidase 4 (also known as CD26) and is involved in a co-stimulatory signal for T-cell receptor-mediated T-cell activation, NF-KB activation and chemokine degradation [48].

In HEK293 cells, *GMPS*, and ten other genes, were down-regulated by the combined treatment (6MP+AP). Interestingly, *GMPS* interacted with the IBD susceptibility candidate gene *CDKAL1* in the network analysis. The protein encoded by *CDKAL1* belongs to a family of methylthiotransferases [49]. In the context of thiopurine metabolism and combination therapy with AP, it is possible that this enzyme has a role in regulating the concentration of methylated thiopurine metabolites.

Translating results from *in vitro* studies to the situation *in vivo* is difficult. However, assuming that proteins encoded by AP regulated genes may interact with other proteins associated with IBD, these genes as well as the interacting genes identified by the two STRING analyses, represent new candidates worth further investigation in the context of combination therapy with thiopurines and AP in IBD-patients. When we investigated the interacting IBD susceptibility candidate genes for enriched GeneOntology terms, purine nucleotide biosynthetic processes were identified at rank 4 in HepG2 cells and at rank 13 in HEK293 cells, and were represented by the genes *ADK*, *ADA*, *ADCY3*, *ADCY7* and *GART*.

Based on its position in the metabolic scheme of thiopurines, blockage of IMPDH could explain a high meTIN/6TGN concentration ratio *in vivo*, and induction of this enzyme by AP could theoretically restore 6TGN. However, the expression of *IMPDH2* was decreased by the combined treatment compared with 6MP alone, whereas the enzymatic activity of IMPDH was unaffected in HEK293 cells.

No effect by the combined treatment (6MP+AP or 6MP+oxypurinol) *vs*. 6MP alone was observed, in any of the studied cell lines, on the absolute concentration of the thiopurine metabolites considered to mediate the cytotoxic effect of 6MP. There are some possible explanations for the lack of effect. Apart from the use of a single cell model, as discussed below, there were large variations in metabolite concentration levels which might have obscured drug-induced changes in concentration. Also, the ratio between methylated and phosphorylated metabolites was low in both cell lines even before AP was added to 6MP. In cell lines with a high mitotic activity, it is also possible that an increased production of 6TGN may be incorporated into DNA [50], and escape detection. In addition, other not yet studied mechanisms may also explain the effects seen *in vivo*.

Even so, we noticed an increase in the metabolite concentration ratio after the combined treatment especially in HepG2 cells without expression of XO, i.e. the same setting that identified the significantly regulated genes in these cells. This observation is the opposite compared to what is seen under combination therapy in RBC *in vivo*. However, this change was not statistically significant after correction for multiple testing in HepG2 cells expressing XO.

To our knowledge there are no previous studies simultaneously investigating the effects of the addition of AP to 6MP on both gene expression levels and thiopurine metabolism in nucleated cells with an active purine *de novo* synthesis. However, the choice of liver cell line to study xenobiotic metabolism has been discussed [24, 26, 30, 51–53]. Here we used HepG2 cells. HepG2 cells and HepaRG cells express many common genes, however, at different levels in comparison with primary human hepatocytes [24, 25, 51, 52, 54]. In relation to RBC, both cell lines, as well as HEK293 cells, are expected to behave differently. As shown in our study, the majority of genes of known relevance to the metabolism of thiopurines are expressed in HepG2 cells and measurable concentrations of thiopurine metabolites were detected. Both the HepG2 and HEK293 cells have previously been used in studies of thiopurine metabolism [24–30, 32]. However, it is well-known that metabolite profiles and the dose-metabolite concentration dynamics may differ between cell lines as well as between blood cells [21, 23, 55–60]. This was illustrated here by the different sensitivities to 6MP as well as the different results noticed on gene expression levels between HEK293 and HepG2 cells. Based on the literature and our observations, we believe it would be difficult to reproduce, in a model based on a single cell line *in vitro*, both the metabolite pattern observed in RBC during monotherapy with 6MP, as well as the effect of combination therapy with 6MP+AP on the metabolite concentration ratio observed in RBC *in vivo*, simply because they represent different biological compartments with different metabolic and transport capacities.

The lack of cytotoxicity data is a limitation of our study. Therefore results should be interpreted with caution. Both apoptosis and oxidative stress are processes closely related to the effects of thiopurine drugs [9, 26, 61, 62]. AP probably has several important roles in thiopurine metabolism, increasing the effect of drug via yet not fully understood mechanisms and by protecting cells from oxidative stress [26, 61]. When AP was combined with thiopurines in HepaRG cells [24], cytotoxicity increased, seemingly mediated by apoptosis/DNA damage at least regarding azathioprine. The concentration of metabolites considered to mediate the cytotoxic effects of thiopurines was however not measured. Even if the experimental settings were not fully comparable, using different cell lines and thiopurine drugs, it is possible that the effects of combination treatment (6MP+AP) on gene expression levels here, in part may be consistent with increased cytotoxicity, especially in the HEK293 cells which were treated with a 6MP concentration corresponding to the IC_50_-value, compared with the nontoxic concentration of 6MP used in HepG2 cells. In HEK293 cells, particularly the down-regulated genes *MGST2*, *IMPDH2*, and *RAC2* interacted with the IBD susceptibility candidate genes associated with the GeneOntology term; apoptotic signaling pathway in response to oxidative stress. It cannot be excluded that a higher concentration of 6MP in HepG2 cells would have resulted in a similar regulation in these cells.

Before choosing the 6MP concentration employed, a small pilot study was conducted in HEK293 cells, which are more sensitive to thiopurine drugs than HepG2 cells [24, 27]. We aimed for a low and as nontoxic concentration as possible but at the same time high enough to generate measurable metabolite concentrations. We noticed a decrease in the concentration of both methylated (from 868 to 93 pmol mg protein^-1^, mean values) and phosphorylated (from 868 to 134 pmol mg protein^-1^) metabolites when cells were treated with 6 μM 6MP *vs*. 3 μM 6MP. Based on the differences in TPMT and HGPRT K_M_ for 6MP [63, 64], it has been suggested that the thiopurine dose reduction, used at combination treatment with AP *in vivo*, in itself may affect the metabolite concentration ratio in RBC [65]. However, no significant effect on the metabolite concentration ratio was observed here. In HepG2 cells both azathioprine and 6MP are expected to be nontoxic up to approximately 300 μM and 4 mM, respectively [24, 26]. We therefore used a concentration of 6 μM, which is not expected to be exceeded in tissue *in vivo* after therapeutic doses [30], in these cells.

Being aware that some glutathione transferases are expressed at low levels in HepG2 cells [24, 25], we used 6MP instead of azathioprine for two reasons; to circumvent the glutathione transferase mediated release of 6MP from azathioprine and to avoid any possible interaction between AP and the released nitroimidazole moiety of azathioprine. This was also supported by a recent study of genetic variants of glutathione transferases in relation to metabolite concentrations in azathioprine treated, and in 6MP treated patients [66].

In summary our results show that the effects of AP in relation to thiopurine metabolism are complex. Previous effects of AP on metabolite concentrations observed in RBC under combined treatment *in vivo* could not be reproduced here in nucleated cells *in vitro*. Given the current understanding of the thiopurine metabolism we find it difficult to generate a data driven hypothesis on APs effects on RBC metabolism based on our *in vitro* results. However, the genes identified indicate that both metabolism and transport may affect the concentration of thiopurine metabolites in cells and their distribution between nucleated cells and RBC. Because of such complex relationships it is possible that metabolite levels in RBC do not necessarily reflect what happens in the drug metabolizing cells. *In vivo* studies in the intestinal mucosa and in hepatic tissue, of genes regulated by the combined treatment as well as their interacting disease associated genes, may further increase our understanding of the mechanisms of AP on the thiopurine metabolism.

## Supporting information

S1 Fig Schematic pathways of azathioprine (AZA) and 6-mercaptopurine (6MP) metabolism. GST, Glutathione transferase; GSH glutathione; XO, xanthine oxidase; AO, aldehyde oxidase; HGPRT, hypoxanthine guanine phosphoribosyltransferase; TPMT, thiopurine S-methyltransferase; SAM, S-adenosyl methionine; IMPDH, inosine 5´-monophosphate dehydrogenase; NAD, nicotine adenosine dinucleotide; ITPase, inosine triphosphatase; GMPS, guanosine monophosphate synthetase; GMP reductase, guanosine monophosphate reductase; GMP kinase, guanylate kinase; RNR, ribonucleotide reductase; NDPK, nucleotide diphosphate kinases; 6-TU, 6-thiouric acid; 6-TIMP, 6-thioinosine monophosphate; 6-TXMP, 6-thioxanthosine monophosphate; 6-TITP, 6-thioinosine triphosphate; meTIMP (or meTIN), methyl thioinosine monophosphate (methyl thioinosine nucleotides); 6-TGMP, 6-thioguanosine monophosphate; 6-TGDP, 6-thioguanosine diphosphate; 6-TGTP, 6-thioguanosine triphosphate; d, deoxy; 6-TGNs, 6-thioguanine nucleotides; PRPP, 5-phosphoribosyl-1-pyrophosphate; PRA, 5-phosphoribosylamine; AMP, adenosine monophosphate.
Figure reprinted with permission from Haglund S, Almer S, Peterson C, Söderman J (2013) Gene expression and thiopurine metabolite profiling in inflammatory bowel disease—Novel clues to drug targets and disease mechanisms? PLOS ONE 8:e56989.
(PDF)

S1 Table Gene expression assays used in HepG2 and HEK293 cells. (DOC)

S2 Table PANTHER over-representation test. (XLSX)
