# Supplementary material for: Combination treatment with 6-mercaptopurine and allopurinol in HepG2 and HEK293 cells – Effects on gene expression levels and thiopurine metabolism
Source: PLoS One. 2017 Mar 9;12(3):e0173825. doi: 10.1371/journal.pone.0173825 (PMC5344510; doi:10.1371/journal.pone.0173825)
Supplement: S1 Table — (DOC) [file pone.0173825.s002.doc]

**S1 Table. Gene expression assays used in HepG2 and HEK293 cells.**

| **Gene symbol** | **Assay ID^a^** | **Description** |
| --- | --- | --- |
| ACTB | Hs99999903_m1 | Actin, beta. Reference gene in HEK293 cells |
| POP4 | Hs00198337_m1 | POP4 homolog, ribonuclease P/MRP subunit. Reference gene in HEK293 cells |
| YWHAZ | Hs00237047_m1 | Protein kinase C inhibitor protein 1. Reference gene in HepG2 cells |
|  | | |
| ABCC4 | Hs00988717_m1 | Multidrug resistance-associated protein 4, ATP-binding cassette, sub-family C, member 4 |
| ABCC5 | Hs00981089_m1 | Multidrug resistance-associated protein 5, ATP-binding cassette, sub-family C, member 5 |
| ALDH1A2 | Hs0018054_m1 | Aldehyde dehydrogenase 1 family, member A2 / Purine metabolism |
| AOX1^b^ | Hs00154079_m1 | Aldehyde oxidase 1, AO |
| CANT1 | Hs00386220_m1 | Soluble calcium-activated nucleotidase 1 |
| CD1D | Hs00939888_m1 | Cd1d molecule |
| CTSS | Hs00175407_m1 | Cathepsin S |
| DEF8 | Hs00276462_m1 | Differentially expressed in FDCP 8 homolog |
| DPP4 | Hs00175210_m1 | Dipeptidyl-peptidase 4 |
| ENTPD1 | Hs00969559_m1 | Ectonucleoside triphosphate diphosphohydrolase 1 |
| ENTPD5 | Hs04176260_g1 | Ectonucleoside triphosphate diphosphohydrolase 5 |
| FAM156A | Hs00739497_s1 | Family with sequence similarity 156, member A |
| FAM46A | Hs00214159_m1 | Family with sequence similarity 46, member A |
| FAR1 | Hs00386153_m1 | Fatty acyl CoA reductase 1 |
| GMPR1 | Hs00199328_m1 | Guanosine monophosphate reductase 1 |
| GMPS | Hs00269500_m1 | Guanosine monophosphate synthetase |
| GNB4 | Hs01118085_m1 | Guanine nucleotide binding protein (G protein), beta polypeptide 4 |
| GSTP1 | Hs02512067_s1 | Glutathione S-transferase pi 1 |
| HPRT1 | Hs99999909_m1 | Hypoxanthine-guanine phosphoribosyltransferase |
| HVCN1 | Hs01032834_m1 | Hydrogen voltage-gated channel 1 |
| IMPDH1 | Hs01597683_g1 | Inosine-5'-monophosphate dehydrogenase 1 |
| IMPDH2 | Hs01021353-m1 | Inosine-5'-monophosphate dehydrogenase 2 |
| ITPA | Hs00738803_m1 | Inosine triphosphatase |
| LAP3 | Hs00429769_m1 | Leucine aminopeptidase 3 |
| MAP3K1_custom | Custom made | Designed based on the chromosomal alignment area of the array probe used in Haglund et al., 2013. |
| MGST2 | Hs00182064_m1 | Microsomal glutathione S-transferase 2 |
| MOCOS^c^ | Hs00215742_m1 | Molybdenum cofactor sulfurase |
| NME1-NME2 | Hs00897135_g1 | Nucleoside diphosphate kinase A and B |
| NME6 | Hs00195083_m1 | Nucleoside diphosphate kinase 6 |
| NT5C1B | Hs00403674_m1 | 5'-nucleotidase, cytosolic IB |
| NT5C2 | Hs01056741_m1 | 5'-nucleotidase, cytosolic II |
| NT5E | Hs01573922_m1 | 5'-nucleotidase, ecto (CD73) |
| NT5M | Hs00220234_m1 | 5',3'-nucleotidase, mitochondrial |
| PGM2 | Hs00217619_m1 | Phosphoglucomutase 2 |
| PLCB2 | Hs01080542_m1 | Phospholipase C, beta 2 |
| PNP | Hs01002926_m1 | Purine nucleoside phosphorylase |
| PPAT | Hs00601264_m1 | Phosphoribosyl pyrophosphate amidotransferase |
| RAC1 | Hs01902432_s1 | Rho family, small GTP binding protein Rac1 |
| RAC2 | Hs01036635_s1 | Rho family, small GTP binding protein Rac2 |
| SLC29A1 | Hs01085706_m1 | Solute carrier family 29 (nucleoside transporters), member 1, equilibrative nucleoside transporter 1 |
| SLC29A2 | Hs00155426_m1 | Solute carrier family 29 (nucleoside transporters), member 2, equilibrative nucleoside transporter 2 |
| SLX1A | Hs02341353_g1 | SLX1 structure-specific endonuclease subunit homolog A, GIY-YIG domain-containing protein 1 |
| SMAP2 | Hs01077385_m1 | Small ArfGAP2, stromal membrane-associated GTPase-activating protein 2 |
| TGOLN2 | Hs00197728_m1 | Trans-Golgi network integral membrane protein 2 precursor |
| TOX4 | Hs00706258_s1 | TOX high mobility group box family member 4 |
| TPMT | Hs00909011_m1 | Thiopurine S-methyltransferase |
| TUSC2 | Hs00200725_m1 | Tumor suppressor candidate 2 |
| UBE2A | Hs00163308_m1 | Ubiquitin-conjugating enzyme E2A |
| XDH | Hs00166010_m1 | Xanthine dehydrogenase/oxidase XO |

*The selected gene expression assays were based on [1]. ^a^Assay identification number according to Life Technologies, Carlsbad, CA, USA. ^b^AOX1; Considered important in the conversion of allopurinol to oxypurinol.^c^MOCOS; Activates the molybdenum cofactor specific for aldehyde oxidase and xanthine oxidase. SMAP2, NT5C1B not analyzed in HEK293 cells.*

1. Haglund S, Almer S, Peterson C and Söderman J (2013) Gene expression and thiopurine metabolite profiling in inflammatory bowel disease - Novel clues to drug tragets and disease mechanisms? *PLOS ONE* **8**:e56989.
